# Pheochromocytoma With High Adrenocorticotropic Hormone Production Capacity Without Pigmentation and Cushingoid Symptoms: A Case Report With a Literature Review

**DOI:** 10.7759/cureus.53358

**Published:** 2024-02-01

**Authors:** Gen Mizutani, Masashi Isshiki, Eisuke Shimizu, Daigo Saito, Akira Shimada

**Affiliations:** 1 Department of Endocrinology and Diabetes, Saitama Medical University, Saitama, JPN

**Keywords:** cushing’s syndrome, 123i-mibg scintigraphy, ectopic acth-producing tumor, catecholamine, pigmentation

## Abstract

Pheochromocytoma or paraganglioma (PPGL) originating from chromaffin cells can produce diverse hormones in addition to catecholamines, including adrenocorticotropic hormone (ACTH). In pheochromocytoma, high levels of ACTH might not result in pigmentation as typically observed in Addison’s disease, and patients might not exhibit the symptoms of Cushing’s syndrome, despite ACTH-dependent hypercortisolism. A 63-year-old male patient with hypertension was admitted to our facility, and computed tomography (CT) revealed a large right adrenal tumor. Despite high plasma ACTH (700-1300 pg/mL) and serum cortisol (90-100 µg/dL) levels, no physical pigmentation or Cushingoid symptoms were observed. Urinary metanephrine and normetanephrine levels reached as high as 16.0 mg and 3.2 mg, respectively. ^123^I-metaiodobenzylguanidine (MIBG) scintigraphy was negative. Low-dose dexamethasone paradoxically increased ACTH and cortisol levels, indicating the potential positive feedback regulation of both hormones by glucocorticoids. The patient was diagnosed with an ACTH-producing pheochromocytoma and underwent successful laparoscopic surgery to remove the adrenal tumor under the intravenous administration of a high-dose α-blocker and hydrocortisone. The levels of ACTH, cortisol, and urinary metanephrine/normetanephrine returned close to normal after tumor removal. We report a rare case of pheochromocytoma with extremely high ACTH/cortisol production but without pigmentation or Cushingoid symptoms. We also reviewed previous reports of ACTH-producing PPGL regarding the paradoxical regulation of ACTH/cortisol by glucocorticoids, pigmentation, Cushingoid symptoms, and negativity of ^123^I-MIBG scintigraphy.

## Introduction

Pheochromocytoma and paraganglioma (PPGL), which are known to result in substantial catecholamine synthesis and release [1], occasionally produce other hormones such as adrenocorticotropic hormone (ACTH) [2]. There is a consensus that ACTH, along with melanocyte-stimulating hormone (MSH), enhances pigmentation and exerts an impact on cardiovascular complications and glucose metabolism by elevating cortisol levels. Cases of hypercortisolism can often complicate the treatment of PPGL and are referred to as ectopic ACTH-producing tumors, a form of Cushing’s syndrome.

We encountered a case of pheochromocytoma with remarkably high ACTH levels but no pigmentation or typical Cushingoid symptoms, along with no uptake of 123I-metaiodobenzylguanidine (MIBG) scintigraphy. The absence of pigmentation led us to review prior reports of ACTH-producing PPGL to investigate the influence of catecholamine excess on ACTH-mediated pigmentation. Furthermore, we conducted an analysis of the prevalence of Cushingoid symptoms, the positivity of MIBG scintigraphy, and the increased incidence of hypertension and diabetes in this condition. The review of this case and past cases is expected to contribute to endocrine clinical practice by enhancing our understanding of the symptoms, diagnosis, and pathophysiology of the rare condition known as ACTH-producing PPGL. Moreover, this report offers a novel clinical perspective on how catecholamine excess affects pigmentation.

## Case presentation

A 63-year-old male was diagnosed with hypertension following an ischemic stroke that occurred five years previously. Despite undergoing multiple antihypertensive treatments, his systolic blood pressure remained elevated at approximately 160 mmHg. Approximately one week prior to hospitalization, the patient experienced a systolic blood pressure exceeding 200 mmHg, with constipation, moderate anorexia, nausea, and vomiting. These gastrointestinal symptoms worsened over several days. Upon hospital admission, abdominal computed tomography (CT) revealed a 6-cm tumor located in the right adrenal gland. To investigate possible endocrine disorders and hypertension, the patient was referred to and immediately hospitalized at our medical facility.

The patient had no medical history other than ischemic stroke and hypertension and no family history of endocrine disorders. His height was 171 cm, his weight was 55 kg, his body mass index was 18.8 kg/m2, and there were no observable Cushingoid symptoms, including moon face, central obesity, or abdominal striae. No hyperpigmentation of the skin or oral mucosa was observed. The patient exhibited transient anuria, dehydration, and hypotension with a blood pressure of 75/54 mmHg. Other vital signs included a body temperature of 36.5 °C, a pulse rate of 96 beats per minute, and an oxygen saturation of 99% in room air. A chest radiograph showed a cardiothoracic ratio of 37% with no lung lesions. Blood tests and urine analysis are shown in Table 1.

**Table 1:** Blood tests and urine analysis

Blood tests		Reference
Blood glucose	132 mg/dL	73–109
Glycated hemoglobin	6.3%	4.6–6.2
Sodium	128 mEq/L	138–145
Chloride	84 mEq/L	101–108
Potassium	3.8 mEq/L	3.6–4.8
Blood urea nitrogen	41.0 mg/dL	8–20
Creatinine	1.10 mg/dL	0.65–1.07
D-dimer	0.92 µg/mL	<1
ACTH	718.7 pg/mL	7.2–63.3
Cortisol	95.2 µg/dL	7.07–19.6
Plasma aldosterone	336 pg/mL	4–82.1
Plasma renin activity	160 ng/mL/h	0.2–2.3
Dehydroepiandrosterone sulfate	496 µg/dL	24–244
Urine analysis		Reference
Metanephrine (MN)	16.00 mg/day	0.04–0.19
Normetanephrine (NMN)	3.20 mg/day	0.09–0.33
Adrenaline	10.10 mg/day	0.0034–0.0269
Noradrenaline	5.10 mg/day	0.0486–0.1684
Dopamine	1.634 mg/day	0.365–0.9615
Free cortisol	4.19 mg/day	0.0043–0.176

**Figure 1:**
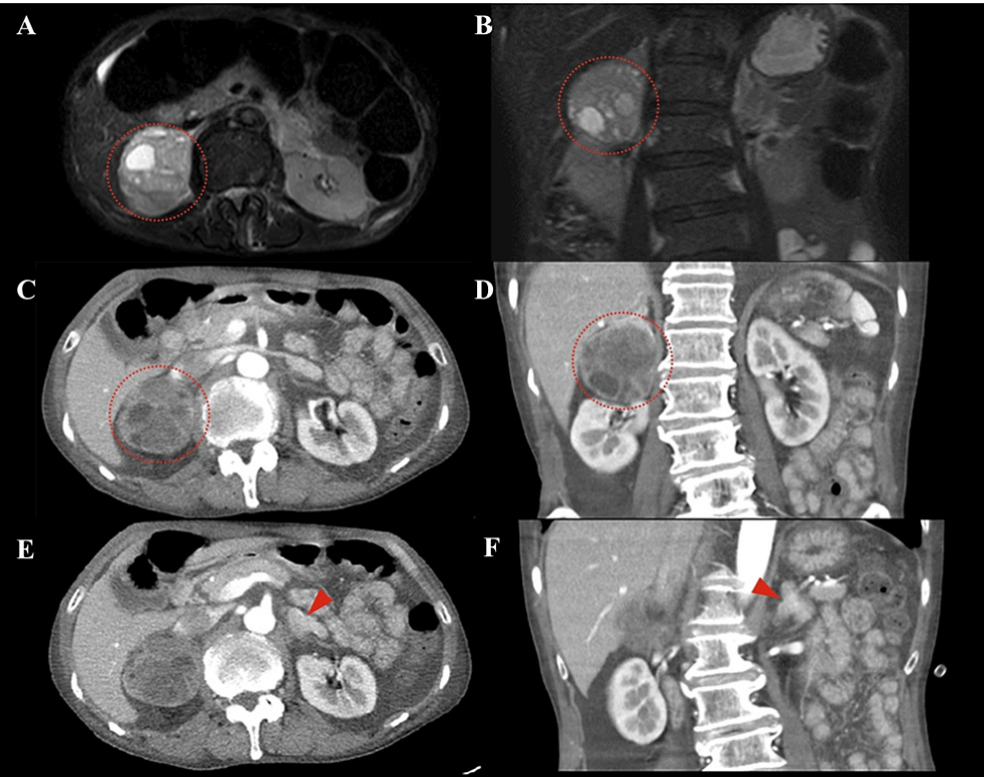
Representative images acquired during the patient’s admission prior to adrenalectomy The images include axial (A) and coronary (B) views from the T2-weighted MRI, along with axial (C, E) and coronal (D, G) views of the enhanced CT scan. Dotted circles and arrowheads indicate the presence of a pheochromocytoma in the right adrenal gland and an enlarged left adrenal gland, respectively.

**Figure 2:**
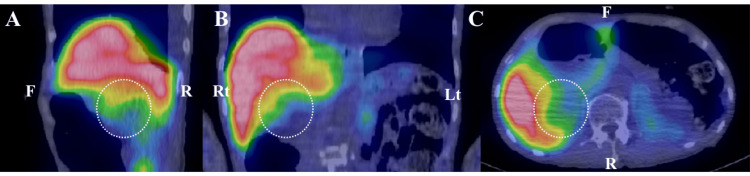
Merged 123I-MIBG scintigraphy expressed in pseudocolors and CT Sagittal (A), coronal (B), and axial (C) views reveal no accumulation in the right adrenal gland (indicated by dotted circles). F, R, Rt, and Lt represent front, rear, right, and left, respectively.

**Figure 3:**
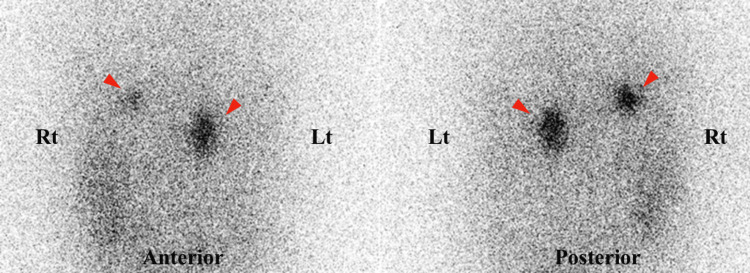
131I-Adosterol scintigraphy Accumulation in both adrenal glands (arrowheads), but exhibiting partial loss of the right adrenal gland. Rt and Lt represent right and left, respectively.

T2-weighted magnetic resonance imaging (MRI) (Figure 1A, 1B) and contrast-enhanced CT (Figure 1C, 1D) revealed a 6-cm right adrenal tumor displaying heterogeneous internal cystic lesions, along with diffuse enlargement of the left adrenal gland (Figure 1E, 1F). The right adrenal gland was displaced upward because of the tumor, but no distinct nodular structure was observed (Figure 1D). Landiolol, a ß-blocker, was introduced in addition to phentolamine to enable the safe performance of a contrast-enhanced CT scan to identify unforeseen vascular structures within the tumor and its periphery, which subsequently confirmed the tumor’s vascular scarcity (Figure 1C, 1D). Scintigraphy using 123I-MIBG revealed no accumulation in the right adrenal tumor (Figure 2), whereas scintigraphy using 131I-adosterol showed bilateral adrenal accumulation with partial signal loss in the lower portion of the right adrenal gland (Figure 3). The pituitary gland was confirmed to be normal on an MRI (data not shown). On the basis of the high levels of urinary catecholamine metabolites, ACTH, and cortisol, as well as imaging studies, we diagnosed this case with right adrenal pheochromocytoma, which might have been concurrently producing ACTH.

Following hospital admission, the patient underwent intravenous fluid administration, leading to a decrease in blood urea nitrogen to 12.4 mg/dL and creatinine to 0.51 mg/dL. Hyponatremia was corrected and normalized to 141 mEq/L. The patient’s blood pressure (systolic) rapidly exceeded 200 mmHg. Consequently, his blood pressure had to be managed by the intravenous administration of nicardipine. Then, nicardipine was switched to phentolamine, an α-blocker, at a dose of 1 µg/kg/min and gradually increased to 15 µg/kg/min before surgery. The constipation gradually improved. To prevent lacunar infarction recurrence, we transitioned from oral clopidogrel to a continuous heparin infusion. Starting from day 8 after admission, heparinization was initiated to target a specific activated partial thromboplastin time range and was continued until six hours before surgery on day 25 as a clopidogrel substitute.

**Figure 4:**
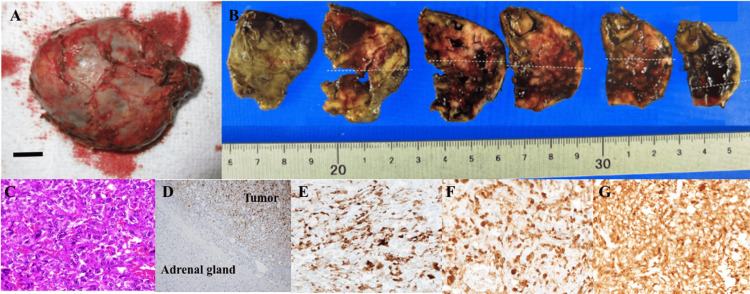
Pathological findings A representation of the entire tumor (A) and images of the tumor’s cut surface (B). (C) The tumor was stained with hematoxylin and eosin and viewed under a magnification of ×400. Immunohistochemistry staining for ACTH at a magnification of ×100 (D) and ×400 (E), along with chromogranin (F) and synaptophysin (G) at a magnification of ×400. Note that image D delineates the interface between normal cortical tissues and tumor tissues visible in the upper-right quadrant. Bar = 1 cm.

**Figure 5:**
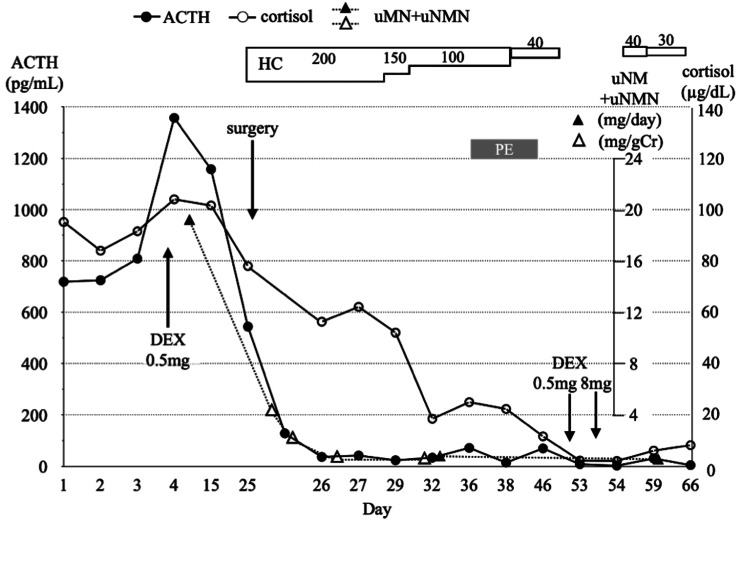
The clinical course of ACTH, cortisol, and total urinary NM+MMN levels The levels of ACTH, cortisol, and total urinary NM+NMN are represented by closed black circles with lines, open circles with lines, and closed (mg/day) or open triangles (mg/gCr) with dashed lines, respectively. Following the surgical procedure, the indicated daily HC infusion (mg/day) was administered for 15 days, with a subsequent transition to oral supplementation, except for days 52 and 53. Note that 0.5 mg dexamethasone (DEX) had a preoperative stimulatory effect on ACTH and cortisol levels rather than suppression, whereas postoperatively, both 0.5 mg and 8 mg DEX suppressed ACTH and cortisol normally (see text and Table 3). The y-axis on the left of the graph corresponds to the level of ACTH, whereas the right y-axis corresponds to the levels of cortisol and urinary NM+NM. Several days after surgery, spot urine NM+NMN/gCr was used as a substitute for the urinary NM+NMN. The gray bar represents an episode of pulmonary embolism (PE).

The patient exhibited no apparent signs of infection, maintained favorable blood pressure control, and remained in stable overall condition. Therefore, metyrapone was deliberately not administered to accurately assess the postoperative treatment effects. A laparoscopic right adrenalectomy was performed successfully after 75 minutes under the uninterrupted administration of phentolamine and hydrocortisone (HC) to control blood pressure and avoid postoperative adrenal insufficiency. The excised tumor had a smooth surface, and upon slicing the tissues, a heterogeneous internal structure was observed, characterized by multiple cysts, areas of hemorrhage, and localized internal necrosis (Figure 4A, 4B). Histological examination of the tumor revealed neoplastic cell growth in the tissue (Figure 4C). As expected, ACTH was distributed in a disseminated pattern throughout the tissue (Figure 4D, 4E). The adrenocortical cells surrounding the tumor showed no ACTH immunostaining. Chromogranin (Figure 4F) and synaptophysin (Figure 4G), the hallmarks of pheochromocytoma, were diffusely and intensely stained, and CD56, an endocrine differentiation marker, was positive (data not shown). The MIB-1 index was 2.2%, and SDHB in tumor cells was recognized by an anti-SDHB antibody (data not shown), indicating it was negative for the SDHx mutation. Additional potential genetic factors, including VHL and RET, were not assessed. The clinical courses of ACTH, cortisol, and the cumulative amount of urinary MN/NMN are shown in Figure 5. Urinary MN/NMN exhibited the most rapid decrease, to approximately 20% of the preoperative level the day after surgery, with a subsequent decline to 1.17 mg/g creatinine four days postoperatively. The ACTH level, which exceeded 500 pg/mL prior to surgical intervention, also promptly normalized within one day of the procedure. Following the normalization of ACTH, the cortisol levels gradually returned to normal over a period of three weeks, likely because of the overestimation of cortisol levels caused by supplementation with HC infusion. Subsequently, a transition was made to the oral administration of HC at a dosage of 40 mg (20 mg in the morning and 20 mg in the evening). The circadian patterns of daily plasma ACTH and serum cortisol profiles on day 3 before surgery were disrupted, and the morning surge of ACTH appeared to recover on day 49 after surgery (Table 2). Before surgery, the oral administration of 0.5 mg dexamethasone (DEX) overnight failed to suppress ACTH and cortisol levels the following morning and resulted in their paradoxical elevation (Figure 5 and Table 3), suggesting the positive feedback of ACTH and cortisol by glucocorticoids. DEX (8 mg) and CRH tests were not conducted because of the patient’s severe illness and the potential exacerbation of his condition by these tests. Two weeks post-surgery, the ACTH levels increased by about 40% in response to 100 µg of CRH. After four weeks, the sequential oral administration of 0.5 mg and 8 mg DEX over two days suppressed ACTH and cortisol levels (Table 3).

**Table 2:** Daily plasma ACTH and serum cortisol profiles on day 3 and day 49 Note that day 3 corresponds to 22 days before surgery, and day 49 corresponds to 24 days post-surgery, during which time the patient was administered 20 mg HC orally twice a day.

Day 3, time	6:00	14:00	23:00	Reference
ACTH (pg/mL)	809.0	842.6	828.6	7.2–63.3
Cortisol (µg/dL)	91.59	95.15	101.00	7.07–19.6
Day 49, time	7:00	14:00	23:00	Reference
ACTH (pg/mL)	69.3	7.4	14.1	7.2–63.3
Cortisol (µg/dL)	11.62	44.70	9.73	7.07–19.6

**Table 3:** Overnight DEX suppression tests pre- and post-surgery, and CRH tests post-surgery Note that tumor removal was performed on day 25. On day 41, the patient was administered 20 mg HC orally twice a day. Supplementation was temporarily discontinued from the evening of day 52 through to day 54.

Suppression test with DEX 0.5 mg			
DEX	Pre (day 3)	0.5 mg (day 4)	Reference
ACTH (pg/mL)	828.6	1357.0	7.2–63.3
Cortisol (µg/dL)	95.2	104.0	7.07–19.6

Suppression test with DEX 0.5 mg and 8 mg				
DEX	Pre (day 52)	0.5 mg (day 53)	8 mg (day 54)	Reference
ACTH (pg/mL)	50.9	7.8	2.2	7.2–63.3
Cortisol (µg/dL)	9.73	2.21	2.05	7.07–19.6

CRH test (day 41)						
Time (min)	0 (pre)	30	60	90	120	Reference
ACTH (pg/mL)	47.2	66.0	61.5	33.4	34.8	7.2–63.3
Cortisol (µg/dL)	7.15	9.06	10.99	7.81	7.57	7.07–19.6

On day 29, the patient resumed clopidogrel four days after surgery. On day 36, the patient experienced chest pain with increased D-dimer levels (9.3 µg/mL). By day 45, the D-dimer levels had peaked at 39.6 µg/mL. A contrast-enhanced CT on day 47 revealed thrombosis in the left femoral vein, popliteal vein, and pulmonary artery in the left lower lobe. Heparinization was resumed on the same day instead of clopidogrel, and a follow-up CT one week later indicated the resolution of the pulmonary artery thrombosis and popliteal vein thrombosis. D-dimer levels improved to 3.8 µg/mL, leading to the replacement of heparin with edoxaban tosylate hydrate (30 mg/day).

## Discussion

**Figure 6:**
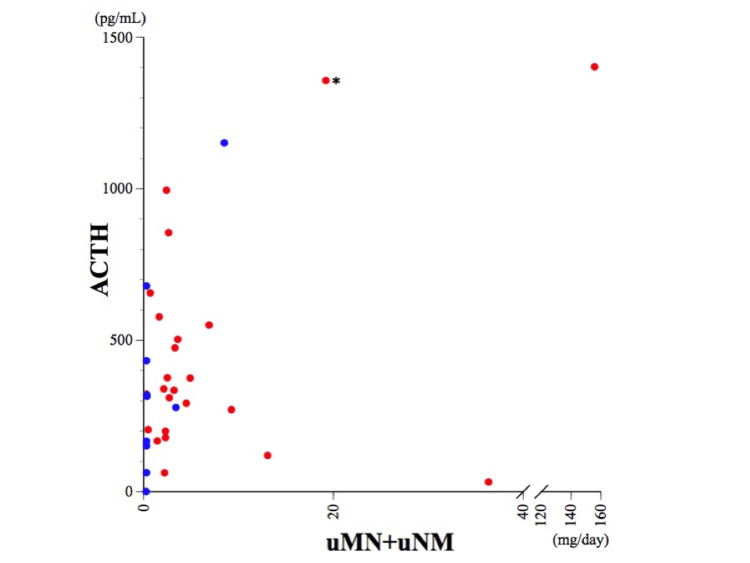
Scatter plot of ACTH and urine MN+NMN Red and blue circles indicate the data of cases with adrenal pheochromocytoma or extra-adrenal paraganglioma, respectively. The asterisk indicates the data of our case. There was no obvious correlation between ACTH levels and total urinary MN and NMN.

We analyzed 60 previous studies of ACTH-producing pheochromocytoma/paraganglioma, comprising 67 cases, as shown in Table 4. This systematic review used the PubMed database to identify English-language manuscripts from 1966 onward, excluding conference abstracts. The search terms were “ACTH-producing pheochromocytoma,” “ACTH-producing paraganglioma,” and “ectopic-ACTH.” We also carefully retrieved additional references from case reports within the review literature that were not captured in the PubMed search procedure. Although most tumors (53 out of 67) originated from the adrenal glands, 14 cases of ACTH-secreting paragangliomas were found to arise from extra-adrenal sites, including the retroperitoneum [3-6], mediastinum [7-9], paranasal region [10-12], lung [13], thymus [14], and cervical and abdominal areas [15,16]. The ages of the patients ranged from 12 to 80 years old, with a mean age of 48.8 years (±14.5 years SD). Three cases were associated with pregnancy [17-19] and three cases had metastasis [6,15,20]. Among 53 adrenal pheochromocytoma cases, 48 were solitary tumors with a left-to-right ratio of 30:18, and five cases were bilateral. Of these five cases, one was conclusively diagnosed as multiple endocrine neoplasia type 2A (MEN2A) [21]. Our findings indicate that female individuals had a significantly higher predisposition for developing ACTH-producing pheochromocytoma/paraganglioma, constituting 73.1% (49 out of 67 cases) of all cases. The maximum diameter of all tumors ranged from 0.8 to 17 cm (mean ± SD = 4.81 ± 2.74 cm). The pheochromocytoma tumor diameter ranged from 1.4 to 10 cm (mean ± SD = 4.51 ± 1.87 cm; median = 4.0 cm, n = 52). The mean ± SD of ACTH, cortisol, and the sum of urinary MN and NMN were 363 ± 318 pg/mL (median = 287 pg/mL, n = 65), 72.8 ± 50.9 µg/dL (median = 61.1 µg/dL, n = 61), and 8.81 ± 26.9 mg/day (median = 2.36 mg/day, n = 34), respectively. No association was detected between ACTH secretion and the combined urinary MN and NMN (Figure 6). These findings imply that the potency of catecholamine and ACTH excretion are not interrelated. In the following analysis, we converted the terms “high ACTH” [8], “normal cortisol” [22], “high cortisol” [22], “cortisol values of x μg/dL or greater”, and “normal urinary MN or NMN” [3,4,12,13,15,23] to numerical values of 70 pg/mL, 12 µg/dL, 22 µg/dL, 1.1 times (110%) of x μg/dL, and 0.3 mg/day, respectively. Additionally, we used the median values to illustrate the range of values and focused on the physical Cushingoid symptoms while excluding hypertension, diabetes, and biochemical changes.

**Figure 7:**
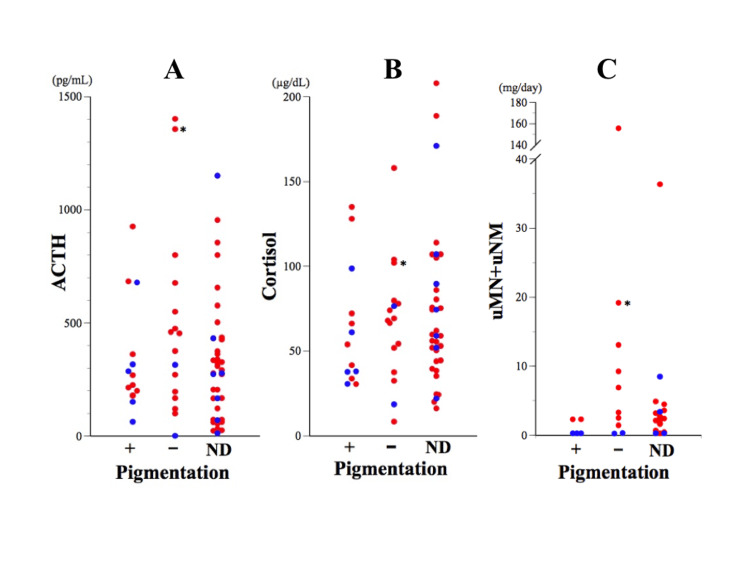
Levels of ACTH (A), cortisol (B), and uMN+uNMN (C) in cases with or without pigmentation ND indicates data for cases without documentation about skin pigmentation. Red and blue dots indicate the data of adrenal pheochromocytoma or extra-adrenal paraganglioma, respectively. The asterisks indicate the data of our case.

The distinct clinical differences between pheochromocytoma and paraganglioma are noteworthy. Although ACTH and cortisol levels were comparable (Figure 7A, B), the combined urinary MN and NMN levels were significantly higher in pheochromocytoma patients than in those with paraganglioma (Figure 6, Figure 7C). Furthermore, most paraganglioma patients exhibited pigmentation (71%, 5 out of 7 cases), whereas pheochromocytoma patients exhibited less frequent pigmentation (35%, 8 out of 23 cases) (Table 4). However, the ratio between the two groups did not reach statistical significance (*p*-value = 0.086, chi-square test). Additionally, the frequency of typical Cushingoid symptoms was similar between paraganglioma (79%, 11 out of 14 cases) and pheochromocytoma (78%, 36 out of 46 cases). Therefore, we deduced the potential pathogenesis of catecholamine hypersecretion in relation to skin pigmentation.

**Table 4:** Clinical characteristics of patients with ACTH-producing PPGL published in the literature Abbreviations: F (female), M (male), HT (hypertension), DM (diabetes mellitus), Pheo (pheochromocytoma), PGL (paraganglioma), I (impaired glucose tolerance), NT (not typical), ND (not documented), NA (not applicable), L (left), R (right), B (bilateral), NS (nasal sinus), RP (retroperitoneum), AG (adrenal gland), MS (mediastinum), PGL (paraganglioma). Data were converted using the following formulas: [cortisol] µg = [cortisol] nmol/L/27.59, [ACTH] pg/mL = [ACTH] pmol/L/0.2202, [MN] g = [MN] × 50.7 mol, [NMN] g = [NMN] × 54.6 mol. *Presence of metastasis in multiple organs. Reference values for the parameters are as follows: uMN: 0.04 to 0.20 mg/day, uNMN: 0.09 to 0.28 mg/day, ACTH: 7.2 to 63.3 pg/mL, and cortisol: 4.5 to 21.1 µg/dL.

Case #	Age (yr)	Sex	HT	DM	Cushingoid symptoms	Pigmentation	uMN (mg/day)	uNMN (mg/day)	uMN+uNMN (mg/day)	ACTH (pg/mL)	Cortisol (µg/dL)	Size (cm)	Location	Pheo or PGL	MIBG	References
1	63	M	+	I	-	-	16	3.2	19.2	1357	104	5	R	Pheo	-	Neumann et al. [1]
2	74	M	+	ND	+	ND	NA	NA	NA	122	55.5	3.5	L	Pheo	NA	Nijhoff et al. [2]
3	55	M	+	-	-	-	0.145	0.117	0.262	1	18.6	17	RP	PGL	NA	Liu et al. [3]
4	53	M	+	+	+	ND	Normal	Normal	Normal	432.4	89.6	4.8	R RP	PGL	NA	Chen, et al. [4]
5	61	F	+	+	+	ND	NA	NA	8.5	1151	171	3	RP	PGL	+	Willenberg et al. [5]
6	12	F	+	+	+	ND	NA	NA	NA	12.5	107.1	10	RP*	PGL	+	Kitahara et al. [6]
7	23	M	+	+	+	+	NA	NA	NA	287	38	ND	Anterior MS	PGL	NA	Flohr and Geddert [7]
8	55	M	ND	ND	NT	ND	NA	NA	NA	High	High	0.8	MS	PGL	NA	Palau et al. [8]
9	51	F	+	+	+	ND	3.4	NA	3.4	278	59	13	Anterior MS	PGL	NA	Park et al. [9]
10	70	F	+	+	NT	ND	NA	NA	NA	273	74.4	ND	L NS	PGL	NA	Thomas et al. [10]
11	68	F	+	+	+	+	NA	NA	NA	317	98.7	4.6	R NS	PGL	NA	Serra et al. [11]
12	50	F	+	+	+	ND	Normal	Normal	Normal	167	52		R NS	PGL	NA	Apple and Kreines [12]
13	39	F	+	ND	+	+	Normal	Normal	Normal	53-73	30.6	6.6	L lung	PGL	NA	Dahir et al. [13]
14	39	M	+	ND	+	+	Normal	Normal	Normal	151.5	37.7	0.8	Thymus	PGL	NA	Li et al. [14]
15	40	F	+	+	+	+	Normal	Normal	Normal	679	61.1	3.5	L kidney*	PGL	NA	Tutal et al. [15]
16	55	F	+	+	+	-	0.19	0.15	0.34	318.4	76.5	5	Near L AG	PGL	+	Otsuka et al. [16]
17	36	F	+	ND	+	ND	0.932	0.706	1.638	577	59.8	3	L	Pheo	-	Langton et al. [17]
18	30	F	+	ND	+	ND	0.29	0.18	0.47	205	56	3.5	L	Pheo	NA	Cohade et al. [18]
19	28	F	+	+	+	ND	0.7	NA	0.7	656	114	4	R	Pheo	NA	Oh et al. [19]
20	80	M	+	+	+	-	76	79.8	155.8	1402	8.4	6.8	L*	Pheo	+	Saishouji et al. [20]
21	29	M	+	+	-	ND	36.2	0.15	36.35	32.5	44.5	10	B	Pheo	+	Moon et al. [21]
22	44	F	+	ND	NT	ND	NA	NA	NA	61	16.2	2.5	L	Pheo	NA	Khalil et al. [22]
23	52	F	+	+	+	ND	Normal	NA	Normal	322	53	4.5	L	Pheo	+	Alvares et al. [23]
24	55	M	+	+	-	+	NA	NA	NA	226	41.7	3.8	R	Pheo	-	Zaman et al. [24]
25	49	F	+	+	-	+	1.48	0.83	2.31	178.7	53.9	4.6	L	Pheo	NA	Krylov et al. [25]
26	51	F	+	+	NT	ND	1.21	1.42	2.63	855	75.7	4	L	Pheo	-	Wan et al. [26]
27	46	F	+	+	+	ND	1.45	0.668	2.118	339.4	107	3.8	L	Pheo	NA	Gabi et al. [27]
28	46	M	+	+	-	-	6.66	2.59	9.25	271	32.5	6	R	Pheo	-	Inoue et al. [28]
29	54	M	+	I	-	ND	NA	NA	NA	23.8	24.57	6.8	B	Pheo	+	Wang et al. [29]
30	50	F	+	+	NT	-	NA	NA	NA	677	>72.5	4.5	L	Pheo	NA	Falhammar et al. [30]
31	44	F	-	-	+	ND	NA	NA	NA	54.5	24.3	5	L	Pheo	NA	Falhammar et al. [30]
32	56	F	+	+	+	ND	1.12	1.29	2.41	995	86	5.4	L	Pheo	+	Sakuma et al. [31]
33	53	F	+	ND	+	ND	3.73	1.17	4.9	375	188.7	4	R	Pheo	NA	Chanukya et al. [32]
34	63	F	+	ND	ND	ND	NA	NA	NA	72.7	80.5	1.4	R	Pheo	NA	Flynn et al. [33]
35	58	M	-	+	ND	ND	NA	NA	NA	205.4	35.3	3.3	B	Pheo	+	Fukasawa et al. [34]
36	75	F	+	+	+	ND	NA	NA	NA	168	75.3	2.5	L	Pheo	NA	Andreassen et al. [35]
37	60	F	+	+	+	ND	NA	NA	NA	427	208	7.5	R	Pheo	NA	Andreassen et al. [35]
38	64	M	+	+	-	ND	NA	NA	NA	363	105	8	L	Pheo	+	Andreassen et al. [35]
39	70	F	ND	+	+	ND	NA	NA	NA	436	62	3	R	Pheo	NA	Folkestad et al. [36]
40	67	F	+	+	+	ND	NA	NA	NA	72.7	38.4	3	R	Pheo	-	Folkestad et al. [36]
41	49	F	+	-	+	-	4.19	2.71	6.9	550	63	4.1	L	Pheo	+	Ballav et al. [37]
42	15	F	+	-	+	+	NA	NA	NA	214.4	33.77	6.5	R multiple	Pheo	NA	Li et al. [38]
43	63	F	+	+	ND	ND	NA	NA	NA	800	298	3.5	R	Pheo	-	Bernardi et al. [39]
44	30	F	+	+	+	ND	NA	NA	NA	62	39.5	3.5	L	Pheo	NA	Ramasamy et al. [40]
45	53	F	+	+	+	ND	NA	NA	NA	327	50.4	3.5	L	Pheo	+	Brenner et al. [41]
46	71	M	ND	+	NT	-	3.3	NA	3.3	475	54.3	4.5	L	Pheo	+/-	Danilovic et al. [42]
47	44	F	+	ND	+	+	NA	NA	NA	926	＞60.2	4	L	Pheo	+	White et al. [43]
48	41	F	-	+	-	-	10.1	2.97	13.07	120	66.5	6	L	Pheo	+	Sato et al. [44]
49	25	F	-	ND	+	-	NA	NA	NA	94.9–105	51.9	4	L	Pheo	NA	Loh et al. [45]
50	51	F	+	+	+	ND	2.2	NA	2.2	62.6	ND	3	L	Pheo	NA	Chen et al. [46]
51	38	F	+	+	+	ND	2.7	NA	2.7	310	ND	2.5	R	Pheo	-	Chen et al. [46]
52	26	M	+	+	+	ND	3.6	NA	3.6	503	ND	3	L	Pheo	-	Chen et al. [46]
53	57	F	+	+	-	ND	4.5	NA	4.5	292	ND	2.8	R	Pheo	-	Chen et al. [46]
54	35	F	+	I	+	-	NA	NA	NA	196	158	2.8	L	Pheo	-	Terzolo et al. [47]
55	49	F	+	+	+	-	NA	NA	1.44	168	74	4	L	Pheo	NA	O’Brien et al. [48]
56	34	M	ND	ND	+	ND	NA	NA	NA	26	20	ND	B	Pheo	NA	Mendonca et al. [49]
57	44	F	+	+	+	-	NA	NA	NA	460	78	9.5	R	Pheo	+	Sakurai et al. [50]
58	36	F	+	I	+	ND	NA	NA	3.2	335	>40	5	R	Pheo	NA	Beaser et al. [51]
59	38	F	+	+	+	+	NA	NA	NA	684	128	6	L	Pheo	NA	Bruining et al. [52]
60	42	F	+	ND	+	-	NA	NA	NA	454	ND	2.8	L	Pheo	NA	Lamovec et al. [53]
61	47	F	+	+	-	+	2.3	NA	2.3	100–300	26–35	7	L	Pheo	NA	Schroeder et al. [54]
62	62	F	+	+	+	-	NA	NA	2.5	376	68	6	R	Pheo	NA	Fiorica et al. [55]
63	35	M	+	-	+	-	NA	NA	NA	NA	37.5	3	L	Pheo	NA	Hoffman et al. [56]
64	47	F	+	+	+	-	NA	NA	NA	800	102	3	L	Pheo	NA	Spark et al. [57]
65	51	F	+	ND	+	+	NA	NA	NA	180	135	8	R	Pheo	NA	Forman et al. [58]
66	67	F	+	+	+	+	NA	NA	NA	176–362	72.2	3	B	Pheo	NA	Berenyi et al. [59]
67	51	F	+	ND	+	ND	1.2–2.2	NA	1.2–2.2	NA	NA	4	R	Pheo	NA	Meloni et al. [60]

Among the cases we examined, our case had significantly elevated ACTH levels, second only to an SDHB-positive malignancy case [20], which resulted in rapid death. Despite the high ACTH levels, our case showed no pigmentation. Our analysis found no significant difference in ACTH and cortisol levels between patients with and without pigmentation (Figure 7A, 7B). Nevertheless, patients lacking pigmentation appeared to have higher catecholamine metabolite levels compared with those with pigmentation (Figure 7C). The means ± SD of total urinary MN and NMN for patients with and without pigmentation were 1.10 ± 1.10 mg/day (median = 0.3 mg/day, n = 5) and 21.2 ± 47.7 mg/day (median = 5.1 mg/day, n = 10), respectively. When the total urinary MN+NMN threshold was set at 2.5 mg, all cases exceeding this value (7 out of 7) lacked pigmentation, whereas 50% of cases below this threshold (4 out of 8) demonstrated pigmentation (Figure 7C). Thus, a reverse relationship seems to exist between high catecholamine production and pigmentation. Hyperpigmentation can be attributed to the excessive secretion of pro-opiomelanocortin (POMC) peptides and α-MSH by the tumor, as well as corticotropin itself [61-64], although the degree of hyperpigmentation was not correlated with the circulating level of α-MSH [65]. Information on the levels of circulating POMC and α-MSH in patients with PPGL is very limited. Despite the reported pigmentation in ACTH-producing pheochromocytoma by White [43], α-MSH levels in that case were undetectable, and it was concluded that the primary cause of pigmentation is likely the ACTH precursor at levels 7-8 times higher than ACTH. Furthermore, compared to Cushing's disease, ectopic ACTH syndrome has been reported to have ACTH precursor levels more than 30 times higher [66]. Although ACTH precursor and α-MSH levels were not measured in this case, it may be that elements other than α-MSH are involved in the pigmentation process in ectopic ACTH syndrome, including ACTH-producing PPGL. In previous reports of 86 cases of Addison’s disease [67] and 16 cases of ACTH-producing tumors other than pheochromocytoma [68], pigmentation was observed in 80 (93%) and 14 (88%) cases, respectively. In our analysis of 67 cases of ACTH-producing PPGL, we assessed the pigmentation status in 31 cases, and only 14 cases (45%) displayed pigmentation. Consequently, patients with ACTH-producing pheochromocytoma seem to have reduced susceptibility to pigmentation compared with those with Addison’s disease or ACTH-secreting ectopic tumors other than pheochromocytoma. One possible explanation for this is that catecholamines may affect the pigmentation process. In mice, the norepinephrine-driven loss of melanocyte stem cells resulted in hair depigmentation [69], and catecholamines inhibited MSH release in mouse ex vivo models through α-adrenergic receptors or dopamine receptors [70,71]; therefore, high levels of catecholamines produced by pheochromocytoma may inhibit the production and release of MSH from POMC or ACTH. Indeed, high catecholamine levels were associated with high human vitiligo [72], and local hyperpigmentation followed autosympathectomy related to a Pancoast tumor [73]. Further investigation is warranted to establish the pathogenetic relationship between high catecholamine levels and skin pigmentation.

**Figure 8:**
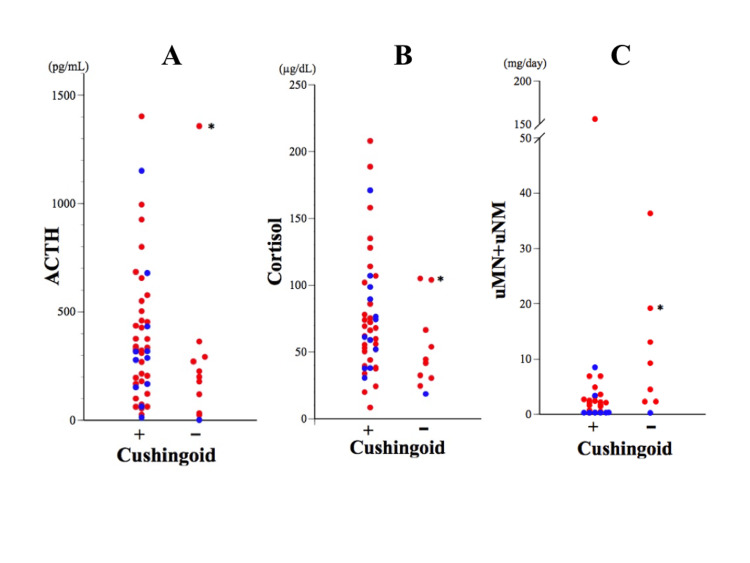
Levels of ACTH (A), cortisol (B), and uMN+uNMN (C) in cases with or without Cushingoid symptoms Red and blue dots indicate the data of cases with adrenal pheochromocytoma or extra-adrenal paraganglioma, respectively. The asterisks indicate the data of our case.

Despite significantly elevated cortisol levels, our case exhibited no Cushingoid symptoms. In our analysis of 65 cases regarding Cushingoid symptoms, 26% (n = 17), including six atypical cases, lacked such symptoms. However, notable differences in ACTH, cortisol, or total urinary MN and NMN were not observed between patients with or without Cushingoid symptoms based on available data (Figure 8A-8C). Ectopic ACTH syndrome typically presents with Cushingoid symptoms in most cases (81-100%), except for pheochromocytoma or small-cell lung cancer [68,74]. Excessive catecholamine production can lead to a hypermetabolic state, resulting in reduced body mass index, body fat content, and inflammatory cytokines [75] and an inverse correlation with weight [76]. The absence of Cushingoid symptoms in our case may be attributed to hypermetabolism induced by extremely high catecholamine levels over a relatively brief period.

ACTH-producing pheochromocytoma promoted a glucocorticoid-driven positive feedback loop on ACTH and catecholamine production. Specifically, metyrapone [20,28,31,57], an 11ß-hydroxylase inhibitor, or adrenalectomy [54] significantly suppressed ACTH and catecholamines, whereas DEX [2,31,57] had the opposite effect. This phenomenon is thought to be mediated by glucocorticoid-induced phenylethanolamine N-methyltransferase and tyrosine hydroxylase and by ACTH production via POMC upregulation [31]. Indeed, our case presented a marked elevation of ACTH levels, even in response to low-dose DEX before surgery, suggesting a glucocorticoid-mediated positive feedback effect on ACTH (Table 3). In contrast, ACTH responded well to CRH two weeks after surgery, and low and high doses of DEX effectively suppressed ACTH four weeks after surgery, implying the restoration of ACTH and cortisol regulation following tumor removal (Table 3). Therefore, it is imperative to avoid a high-dose DEX suppression test in PPGL patients exceeding 2 mg due to the risk of hypertension crises. Furthermore, even a low-dose DEX test requires careful execution when considered indispensable, as demonstrated in our study. Additionally, the potential use of metyrapone to manage urgent hypercortisolism and catecholamine crises should be considered.

Despite notable catecholamine synthesis, the MIBG uptake in our case of pheochromocytoma was negative. According to a recent review, the sensitivity of MIBG scintigraphy to detect PPGL, including extra-adrenal, multiple, recurrent, and hereditary cases, ranged from 52% to 75% [77]. In our review, the MIBG positivity rate for ACTH-producing PPGL was 63% (15 out of 27 patients), in alignment with this range. Another study indicated that MIBG uptake was correlated with the size of the tumor and epinephrine production [78]. Specifically, in ACTH-producing PPGL cases that were MIBG-negative, the average tumor size was significantly smaller (mean 3.6 cm with a SD of 1.01, n = 11) compared with MIBG-positive cases (mean 6.0 cm with a SD of 2.4, n = 15; p = 0.006, t-test). False-negative results are common in SDHB-related pheochromocytoma with high metastatic potential [79] and in cases with RET gene mutations [80]. Other factors leading to false-negative results include hemorrhage or necrosis in cystic lesions [81,82], a lack of VMAT-1 expression [83], and certain medications, including ß-blockers [84]. In our case, the lack of MIBG uptake could be attributed in part to necrotic changes within the tumor and the concurrent use of landiolol, a medication that can reduce MIBG uptake.

During PPGL, excessive catecholamines can lead to various cardiovascular conditions, which can sometimes be fatal, including cardiomyopathy [85]. These cardiomyopathies occurred in 11% of PPGL cases [86]. Corticosteroids, among various medications, can exacerbate cardiovascular complications in PPGL [87]. Notably, the prevalence of diabetes (94%, 59 out of 63 cases) and hypertension (92%, 48 out of 52 cases) in ACTH-producing PPGL was higher than in cases with Cushing’s syndrome alone [88] or pheochromocytoma alone [89, 90]. Therefore, ACTH-producing PPGL, which increases the levels of cortisol and catecholamines, is considered to increase the risk of cardiovascular, immune, metabolic, and psychological complications [2,20].

## Conclusions

In summary, we report a case of pheochromocytoma with remarkably elevated ACTH/cortisol production and paradoxical responses to glucocorticoids, despite the lack of pigmentation and Cushingoid symptoms, as well as the lack of uptake of 123I-MIBG. Furthermore, we conducted a literature review of ACTH-producing pheochromocytoma, focusing on the increased comorbidity of hypertension and diabetes, the paradoxical regulation of ACTH/cortisol by glucocorticoids, pigmentation, Cushingoid symptoms, and negative 123I-MIBG scintigraphy results.
